# Treatment of Anterior Cruciate Ligament Injury in Skeletally Immature Patients

**DOI:** 10.1155/2012/932702

**Published:** 2012-01-18

**Authors:** Joshua L. Hudgens, Diane L. Dahm

**Affiliations:** Department of Orthopaedic Surgery, Mayo Clinic, 200 First Street SW, Rochester, MN 55905, USA

## Abstract

The incidence of ACL tears is rising in the pediatric and adolescent populations as these individuals succumb to traumatic and nontraumatic athletic injuries. Management of this condition in the skeletally immature patient poses a challenge and is controversial. Operative reconstruction carries the concern for damage to the physis with resultant limb length inequality and angular joint deformity but provides stability to the knee and allows return of function in most patients. On the other hand, nonoperative treatment has been shown to carry an increased risk of meniscal and articular cartilage damage and is difficult from a compliance standpoint in this demographic. For the majority of skeletally immature patients, operative treatment is recommended as it has shown good clinical and functional results with minimal risk of growth disturbance. This paper aims to address the natural course of ACL injuries in the skeletally immature patient, treatment options with associated complications, and current preventative strategies.

## 1. Introduction

The treatment of anterior cruciate ligament (ACL) injuries has spawned a great deal of research. However, the majority of the literature on the topic deals with adults. Far fewer studies have focused on the management of this condition in the pediatric and adolescent patient. Consequently, the management strategy for ACL injuries in this population is not as clearly elucidated as it is for their skeletally mature counterparts.

Children are sustaining injuries to the ACL at an increasing frequency. Shea et al. [1] looked at insurance claims over a five-year period for soccer players aged 5 to 18 years and noted knee injuries constituted 22% of all injuries, while ACL injuries accounted for 31% of the total knee injury claims. The risk for complete ACL tears rises as children mature secondary to increased skeletal rigidity [2]. Conversely, tibial spine avulsions and partial ACL tears constitute a greater percentage of total ACL injuries in preadolescents than in older children, due to the ability of the immature skeleton to absorb force at this age. Nonetheless, complete ACL tears comprise a major portion of ACL injuries in younger children [1–3]. Kellenberger and Von Laer [4] similarly noted in a review of 330 patients with knee injuries that 80% with tibial eminence avulsions were less than 12 years old, whereas 90% of the patients with nonosseous ACL lesions were over 12. From a gender standpoint, it has been shown that as females approach skeletal maturity, and certainly upon reaching it, they have a higher risk of ACL rupture (two to eight times) than do males. However, while still skeletally immature, the reverse is true with boys having the higher incidence of the two sexes [2, 5].

Historically, significant debate regarding the proper management of ACL injury in the skeletally immature patient has existed. Two basic options are available, surgical reconstruction and conservative management, each with their own potential sequelae. Theoretically, there has been concern that operative management would violate the growth plate resulting in concomitant growth disturbance and angular or rotational deformity of the limb [6–10]. Consequently, many patients have been treated conservatively via activity modification and bracing with postponement of surgery until skeletal maturity [11–13]. Yet conservative treatment is not without risk. It has been shown that 21–100% of children sustaining ACL injuries will have coexistent meniscal damage [11, 14–18]. Additionally, this age group is poorly compliant with activity restrictions leading to a significant risk of further meniscal injury or articular cartilage damage [11, 15, 18, 19]. In a recent systematic literature review, Vavken and Murray [20] identified only 1 study with level II evidence and 10 with level III evidence on this topic. This understates the need for further prospective studies on the subject.

This paper aims to discuss ACL tears in the skeletally immature patient, specifically focusing on the natural history, nonoperative management, operative management including complications, and prevention of these injuries.

## 2. Natural History/Nonoperative Management

In the pediatric and adolescent patient, the location of the ACL injury is an important determinant of management. Common in this population, tibial spine avulsion injuries, if nondisplaced, can be treated nonoperatively with satisfactory outcomes. However, displaced avulsion injuries require arthroscopic reduction and internal fixation [21, 22]. Similarly the extent of the ACL tear is important to differentiate. Nonsurgical management of partial tears may yield acceptable results in this population when paired with a structured rehabilitation program. However, children and adolescents with greater than 50% tears of the ACL have been shown to have poor outcomes if not surgically reconstructed and may progress to a complete tear [23].

For complete tears of the ACL, nonoperative treatment generally carries an unfavorable prognosis. It has been shown in numerous studies to lead to increased intra-articular damage in the form of meniscal tears and damage to the cartilage [11, 12, 15, 16, 24]. In a study of 39 pediatric and adolescent patients with an average age of 13.6 years at injury, Millett et al. retrospectively compared acute surgical reconstruction (less than 6 weeks from time of injury), to chronic reconstruction (more than 6 weeks after injury). A highly significant relationship was found between time to surgery and medial meniscal tears. 36% of patients in the chronic group sustained medial meniscal tears versus only 11% of acute group, which led the authors to support early operative intervention [18]. Henry et al. [19] retrospectively looked at 56 patients who sustained an ACL tear while skeletally immature and compared those who had open physes at reconstruction with those whose reconstruction was delayed until skeletal maturity. Mean time to surgery was 13.5 and 30 months, respectively. A higher rate of medial meniscal tears in the delayed group, (41%) compared to the open physes group (16%) was noted. In addition, a higher rate of meniscectomy and lower subjective outcome scores were noted in the delayed group. No growth disturbances were found in either group. Also, in a recent comparative study, Streich et al. [25] compared 28 children with intraligamentous tears of the ACL, of which 12 were treated nonoperatively and 16 operatively. All were Tanner stage 1 or 2 with a mean age of 11 years at time of treatment. Interestingly, the surgery group was selected to include only patients who had concomitant damage to the meniscus or articular cartilage. The nonoperative group included only isolated ACL ruptures. At a mean follow-up time of 70 months, the patients had grown an average of 20.3 cm with no evidence of leg length inequality or angular deformity in either group. However, the surgical group had significantly better clinical and functional results than did the nonoperative group. Additionally, 58% of the nonoperatively managed patients went on to require surgical intervention due to persistent instability.

Few studies have shown conservative management to be a viable treatment option. A recent systematic review found only 1 study that showed no increase in secondary intra-articular injury in conservatively treated patients in whom surgery was delayed until skeletal maturity [20]. The identified study by Woods and O'Connor [13] retrospectively compared two groups of adolescents with ACL rupture. One group of 13 adolescents with a mean age of 13.8 years at time of injury, presented with open physes. Surgery was delayed until skeletal maturity and performed at a mean of 70 weeks following injury. The other group of 116 adolescents had a mean age of 15.0 years at time of injury, presented at various time intervals after ACL rupture, and were skeletally mature on presentation. The skeletally mature group was not intentionally delayed and had a mean time interval from injury to surgery of 14.1 (0.3–355.1) weeks. No significant difference with respect to overall additional knee injuries, meniscal injuries, and articular cartilage injuries was noted between the delayed patients and the skeletally mature patients. The authors attributed the lack of additional knee injuries in the delayed group to strict adherence to nonoperative treatment including, complete abstention from sports activities and daily use of an ACL brace. One self-described limitation of this study was a lack of statistical power due to small sample size.

Another study by Moksnes et al. [26] examined ACL rupture in children 12 years of age and younger comparing 20 nonoperatively treated patients to 6 delayed reconstruction patients at a minimum of 2-year postinjury or postoperative followup respectively. Patients were classified as “copers,” if they had returned to preinjury activity level and performed above 90% in all hop tests, or as “noncopers.” Of the nonoperative group 65% returned to preinjury activity level and 50% were classified as “copers” at followup. Only 9.5% of the nonoperative group suffered secondary meniscal injury. Based on the large number of “copers” in the nonoperative group and relatively low number of meniscal injuries, a treatment algorithm based on functional and patient subjective measures was suggested that could identify patients who could be allowed to participate in their desired activities until skeletal maturity when ACL reconstruction could be considered.

Due to the substantial amount of literature showing risk of further damage to the joint and recurrent instability requiring surgical intervention, prolonged nonoperative therapy for complete ACL rupture remains controversial. In addition, from a compliance standpoint, the pediatric and adolescent population will likely have significant difficulty with stringent activity restrictions. However, if nonoperative treatment is chosen, the protocol should include bracing of the affected knee, restriction of sports participation and other activities involving jumping and pivoting, and structured physical therapy and rehabilitation [27].

## 3. Operative Management

### 3.1. Surgical Anatomy

The ACL functions as the primary restraint to anterior tibial translation. It traces a course from its proximal attachment on the posteromedial portion of the lateral femoral condyle and travels distally and medially to attach in front of and lateral to the tibial spine on the anterior tibia [28, 29]. Though the ACL is proportionally smaller in the pediatric and adolescent populations compared to the adult population, the anatomic landmarks remain the same [30]. ACL reconstruction aims to restore stability and functionality to the knee by recreating the native anatomy.

The primary concern with ACL reconstruction in the skeletally immature patient is disruption of the tibial or femoral physis with resultant growth disturbance and deformity of the joint. Approximately two-thirds of the length of the lower extremity is derived from growth at the knee joint, specifically from the distal femoral and proximal tibial physes. The distal femoral growth plate is actually the largest and fastest growing physis in the human body accounting for roughly 70% of the length of the femur and 40% of the length of the entire lower limb [31]. Similarly the proximal tibial growth plate contributes 55% of tibial length and 25% of leg length. On average, the two growth plates add approximately 1 cm and 0.6 cm of length, respectively, to the lower extremity per year. They do so until final skeletal maturation takes place, usually between age 14–16 years in girls and 16–18 years in boys [31, 32].

### 3.2. Transphyseal Reconstruction

The transphyseal technique for ACL reconstruction is the standard operative method for treating adult patients. Consequently, when adolescents are nearing skeletal maturity, it is commonly accepted that they may be managed as adults. McCarroll et al. [33] reported good to excellent results in a cohort of 60 athletes with a mean age of 14.2 years, using transphyseal ACL reconstruction with bone-patellar tendon-bone (BTB) graft. Of note, BTB grafts are typically avoided in the skeletally immature patient as growth arrest can be induced from bone bridges resulting from insertion of the bony portion of the graft across the physis. For this reason, Kocher et al. [34] advocated the use of soft-tissue grafts in ACL reconstruction of skeletally immature pubescent adolescents. In their study of 59 patients with a mean age of 14.7 years, excellent functional results were reported with a low revision rate and minimal growth disturbance using transphyseal ACL reconstruction with autogenous hamstring grafts.

Transphyseal techniques have shown satisfactory results even in less mature patients. Mcintosh et al. described good clinical results and return to previous activity level in patients with wide open physes who had undergone transphyseal reconstruction. Even in Tanner stage 1 or 2 patients, two studies have shown satisfactory results with transphyseal procedures in patients with a mean age of 12.1 years and 11 years, respectively [25, 35]. No growth disturbance was noted in either study, and only one patient was noted to have an angular deformity, which was not deemed to cause any functional impairment. When utilizing transphyseal techniques in these patients, of paramount importance is the avoidance of fixation devices or hardware crossing the physis.

### 3.3. Physeal Sparing Reconstruction

As an alternative to the transphyseal approach, a number of physeal sparing techniques, both intra-articular and extra-articular, have been described. Theoretically, these techniques should minimize the risk of growth disturbance or angular deformity by avoiding violation of the physis. Though a number of retrospective studies exist with the majority achieving excellent results, there is a scarcity of prospective or comparative data that would advocate the superiority of one method over the other. One of the first to use a physeal sparing approach, Macintosh and Darby [36] in 1976 described good results using a portion of the iliotibial band looped around the lateral femoral condyle, through the knee and attached to the proximal tibial metaphysis distally to reconstruct the ACL. This technique has been modified by others for use in the skeletally immature patient. A recent systematic review identified 6 studies using modifications of this physeal sparing, extraosseous reconstruction technique, and showed no growth deformity at an average 47.3 month followup in patients with a mean age of 12.1 years [20].

An all-epiphyseal technique was described by Guzzanti et al. [37] in which the tunnels were drilled through the distal femoral and proximal tibial epiphyses. 5 preadolescents (Tanner stage 1) at a minimum of 4-year followup demonstrated excellent stability and no leg length discrepancy or angular deformity. Other studies have also shown all-epiphyseal techniques to be safe and efficacious [38, 39]. A hybrid of physeal sparing and transphyseal approaches, partial transphyseal techniques utilize only one tunnel through the physes thereby limiting, in theory, the possibility for growth disturbance. Several studies utilize this method, which has been described both with tunnels drilled only through the femoral epiphyses and with tunnels drilled only in the tibia. Both techniques have demonstrated satisfactory results [14, 17, 40, 41].

## 4. Complications of Operative Management

Growth disturbance and angular deformity after ACL reconstruction in the skeletally immature patient have been a primary area of concern for surgeons treating patients in this demographic. In animal studies, various technical factors have been associated with physeal injury and subsequent growth disturbance, including fixation of the graft near or across the physis [6], increased tunnel diameter in relation to physeal diameter [7, 9], overtensioning of the graft [6, 42, 43], placement of bone blocks across the physis [44], inadequate filling of the tunnels with graft material [45, 46], and tunnel malposition [47]. An extensive discussion of these technical aspects is beyond the scope of this review. In a survey of The Herodicus Society and The ACL Study Group, Kocher et al. [8] identified 15 reported cases of growth disturbance/angular deformity in human patients. The main factors associated with these cases were hardware fixation across the physis and spanning the physis with graft bone plugs. Large tunnel size was also associated with these undesirable outcomes. Though growth deformities have been clearly demonstrated in scientific studies using animal models, the vast majority of these reports in skeletally immature humans are from case studies and survey data [8, 48, 49]. Vavken and Murray [20] in their recent systematic review of ACL tears in the skeletally immature patient identified 31 studies (479 total patients) of ACL reconstruction with at least 1 transphyseal tunnel and noted only 3 angular deformities and 2 limb-length discrepancies. They identified 6 reports (106 total patients) of extraphyseal reconstruction with no growth deformities described. In comparison, the same systematic review identified 12 articles (476 total patients) reporting nonoperative management with a mean of 50.2% of patients who required later surgical stabilization due to unstable knees with severe meniscal and cartilage damage.

When comparing operative techniques, few studies actually contrast the various operative procedures. In a recent review, Kaeding et al. [50] identified 13 case series of various ACL reconstruction methods in 187 Tanner stage I, II, and III patients. They concluded that there was no clinical difference between transphyseal and physeal sparing techniques as both produced excellent clinical results in Tanner stage II and III patients with a very low incidence of growth abnormalities in either group. Due to a lack of studies in Tanner stage I patients, no firm conclusions could be reached regarding this patient subset. Frosch et al. [51] recently performed a meta-analysis of operative treatment in the skeletally immature patient. This included 55 studies and 935 patients with a mean age of 13 years, and a mean followup of 40 months. They determined the rate of leg-length discrepancy or axis deviation to be 1.8% (95% confidence interval [CI], 0% to 3.9%). Also of note, transphyseal techniques were associated with a lower risk of leg-length discrepancy or axis deviation than were physeal sparing procedures (1.9% versus 5.8%; relative risk, 0.34; 95% CI, 0.14 to 0.81). The authors theorized that this phenomenon may have resulted from drilling close to the growth plate in the physeal sparing techniques potentially leading to heat damage and early closure.

## 5. Prevention

Though there may be little that can be done to prevent traumatic (contact) ACL injuries, discrete risk factors do exist for noncontact ACL tears [52]. Specifically, neuromuscular, anatomical, hormonal, shoe-surface interaction, and environmental causes have been identified as potential risk factors for ACL rupture [53]. Many current preventative strategies center around neuromuscular training programs. Mandelbaum et al. [54] conducted a prospective non-randomized controlled trial over the course of two years in female soccer players aged 14–18 years. They examined the effectiveness of a neuromuscular and proprioceptive training program, including education, stretching, strengthening, plyometrics, and sports-specific agility drills, on the incidence of ACL injuries and noted a significant decrease in the treatment group over the two year period. Interestingly, Distefano et al. [55] conducted an ACL prevention study on 65 soccer athletes with a mean age of 10 years. They compared pediatric specific and traditional training programs to non-trained controls with the hypothesis that the age specific program would produce the best results. However, the youth athletes saw no significant improvement versus the controls in the pediatric program, whereas significant improvements in balance and vertical jump height were noted with the traditional program.

From a training standpoint, multicomponent prevention programs have shown better noncontact injury reduction results [52]. Specific training areas include balance, lower extremity strength and stretching, body awareness, and core strength and control. These facets of a neuromuscular program work to reduce risk factors such as landing force and knee valgus moments as well as increasing advantageous, protective muscle activation [52]. However, further research is required in order to validate the effectiveness of any proposed intervention program.

## 6. Conclusion

Managing ACL tears in the skeletally immature patient is a complicated and at times challenging undertaking. As such, it should be undertaken only by a surgeon with experience treating pediatric and adolescent injuries of this nature. Two basic choices exist: (1) conservative management with or without delayed reconstruction or (2) early reconstruction. While data can be found to support both modes of care, an overwhelming preponderance of the literature supports early operative intervention for complete ACL tears in this population. Operative intervention has consistently been shown to increase knee stability and decrease the risk of further damage to the meniscus and articular cartilage with minimal risk of growth disturbance. Conservative or delayed operative care should only be considered in the most compliant patients with uncomplicated injuries. As there is little data supporting one surgical technique as superior, patient age and surgeon familiarity and comfort should guide the choice.
